# In Vitro Efficacy of Dalbavancin as a Long-Acting Anti-Biofilm Agent Loaded in Bone Cement

**DOI:** 10.3390/antibiotics12091445

**Published:** 2023-09-13

**Authors:** Mar Sánchez-Somolinos, Marta Díaz-Navarro, Antonio Benjumea, José Matas, Javier Vaquero, Patricia Muñoz, Pablo Sanz-Ruíz, María Guembe

**Affiliations:** 1 Department of Clinical Microbiology and Infectious Diseases, Hospital General Universitario Gregorio Marañón, 28007 Madrid, Spain; mssomolinos@salud.madrid.org (M.S.-S.); marta.diaz@iisgm.com (M.D.-N.); 2 Instituto de Investigación Sanitaria Gregorio Marañón, 28007 Madrid, Spain; jvaquero@salud.madrid.org (J.V.); pmunoz@hggm.es (P.M.); psanzr@salud.madrid.org (P.S.-R.); 3 Department of Orthopaedic Surgery and Traumatology, Hospital General Universitario Gregorio Marañón, 28007 Madrid, Spain; antonio.benjumea@salud.madrid.org (A.B.); joseantonio.matas@salud.madrid.org (J.M.); 4 School of Medicine, Traumatology Department, Universidad Complutense de Madrid, 28040 Madrid, Spain; 5 School of Medicine, Microbiology Department, Universidad Complutense de Madrid, 28040 Madrid, Spain; 6 CIBER Enfermedades Respiratorias-CIBERES (CB06/06/0058), 28029 Madrid, Spain

**Keywords:** dalbavancin, bone cement, polymethylmethacrylate, elution, biofilm, vancomycin

## Abstract

Based on previous studies by our group in which we demonstrated that dalbavancin loaded in bone cement had good elution capacity for the treatment of biofilm-related periprosthetic infections, we now assess the anti-biofilm activity of dalbavancin and compare it with that of vancomycin over a 3-month period. We designed an in vitro model in which we calculated the percentage reduction in log cfu/mL counts of sonicated steel discs contaminated with staphylococci and further exposed to bone cement discs loaded with 2.5% or 5% vancomycin and dalbavancin at various timepoints (24 h, 48 h, 1 week, 2 weeks, 6 weeks, and 3 months). In addition, we tested the anti-biofilm activity of eluted vancomycin and dalbavancin at each timepoint based on a 96-well plate model in which we assessed the percentage reduction in metabolic activity. We observed a significant decrease in the dalbavancin concentration from 2 weeks of incubation, with sustained anti-biofilm activity up to 3 months. In the case of vancomycin, we observed a significant decrease at 1 week. The concentration gradually increased, leading to significantly lower anti-biofilm activity. The percentage reduction in cfu/mL counts was higher for dalbavancin than for vancomycin at both the 2.5% and the 5% concentrations. The reduction in log cfu/mL counts was higher for *S. epidermidis* than for *S. aureus* and was particularly more notable for 5% dalbavancin at 3 months. In addition, the percentage reduction in metabolic activity also decreased at 3 months in 5% dalbavancin and 5% vancomycin, with more notable values recorded for the latter.

## 1. Introduction

Prosthetic joint infection (PJI) is a complication that occurs in approximately 1%–2% of patients undergoing prosthetic surgery and is related to high morbidity and mortality rates and health care costs [1,2,3].

Given that the main causative factor associated with PJI is the ability of microorganisms to form a biofilm on the surface of the prosthesis, eradication is difficult [4,5,6]. Conventional management is based on radical debridement and single-stage, two-stage, or even resection arthroplasty combined with various antibiotic protocols [7,8,9,10].

The most commonly used antibiotic bone cement in two-stage procedures is polymethylmethacrylate (PMMA), owing to the wide experience in its use and its favorable safety profile [11,12]. Given the low number of antibiotics recommended for loading in bone cement, we recently reported, for the first time, that dalbavancin was a suitable alternative in bone cement based on its good elution capacity over a 14-day in vitro study period [13]. However, it is necessary to assess whether elution is maintained over longer periods and whether eluted dalbavancin can reduce staphylococcal biofilm. It is particularly important to compare this agent with vancomycin, which is one of the most widely used antibiotics loaded in bone cement and belongs to the same antibiotic group as dalbavancin (lipoglycopeptides).

Therefore, we aimed to assess the elution capacity and the anti-biofilm activity of dalbavancin and to compare it with that of vancomycin, both at concentrations of 2.5% *w*/*w* and 5% *w*/*w*, over a 3-month period.

## 2. Results

### 2.1. Antibiotic Concentrations

**Figure 1:**
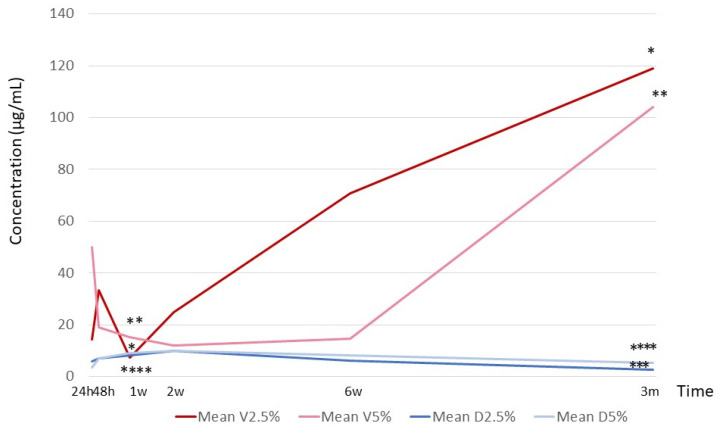
Concentration of antibiotic release over a 3-month period for vancomycin (V) and dalbavancin (D); h, hours; w, weeks; and m, months. A significant decrease (µg/mL) was observed between the median concentration of 2.5% vancomycin at 24 h vs. 1 w (*p* = 0.05, *), 24 h vs. 3 m (*p* = 0.05, *), 2 w vs. 3 m (*p* = 0.05, *), and 1 w vs. 3 m (*p* = 0.05, *). A significant decrease was observed between the median concentration of 5% vancomycin at 24 h vs. 1 w (*p* = 0.037, **), 24 h vs. 3 m (*p* = 0.05, **), 2 w vs. 3 m (*p* = 0.05, **), and 1 w vs. 3 m (*p* = 0.037, **). A significant decrease was observed between the median concentration of 2.5% dalbavancin at 1 w vs. 3 m (*p* = 0.05, ***) and 2 w vs. 3 m (*p* = 0.05, ***). A significant decrease was observed between the median concentration of 5% dalbavancin at 24 h vs. 1 w (*p* = 0.05, ****), 1 w vs. 3 m (*p* = 0.05), and 2 w vs. 3 m (*p* = 0.05, ****).

Although the concentration of vancomycin decreased significantly at 2 weeks, it gradually increased significantly at both the 2.5% and the 5% formulations. In contrast, dalbavancin reached its highest concentration at 2 weeks before gradually decreasing at both concentrations used (Figure 1).

### 2.2. Anti-Biofilm Activity of Eluted Antibiotics

**Figure 2:**
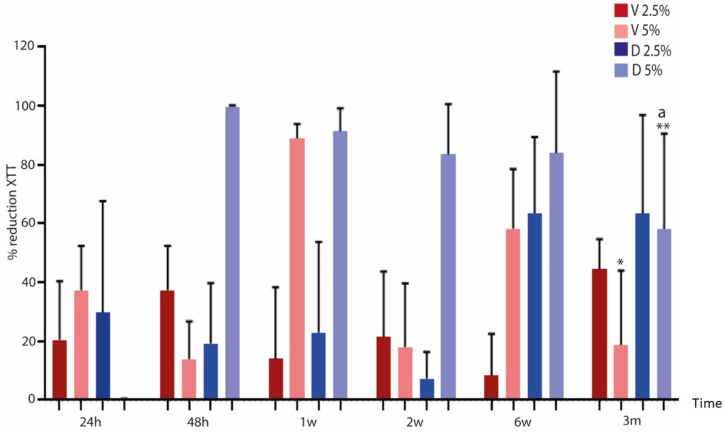
Overall mean percentage reduction in metabolic activity of 24 h biofilms formed on 96-well plates treated with each antibiotic elution over a 3-month period. V, vancomycin; D, dalbavancin; h, hours; w, weeks; and m, months. * A significant decrease was observed between median XTT percentage reduction in 5% vancomycin at 24 h vs. 3 m (*p* = 0.044) and 1 w vs. 3 m (*p* < 0.001). ** Significant decrease between median XTT percentage reduction in 5% dalbavancin at 1 w vs. 3 m (*p* = 0.044). ^a^ Significant decrease for median XTT percentage reduction between 5% vancomycin and 5% dalbavancin at 3 m (*p* = 0.044).

Overall, we observed a significant decrease in the median (IQR) percentage reduction in metabolic activity at the end of the study period (3 months) with respect to 24 h and 1 week for 5% vancomycin (8.7% [0.0%–47.5%] vs. 42.6% [20.2%–49.1%] and 90.5% [83.6%–93.0%], *p* = 0.0044 and *p* < 0.001, respectively). A significant decrease was also observed in the median (IQR) percentage reduction in metabolic activity at 3 months with respect to 1 week for 5% dalbavancin (43.9% [35.4%–95.3%] vs. 89.3% [84.1%–100%], *p* = 0.044) (Figure 2).

Regarding differences between both antibiotics, a statistically significant decrease in median (IQR) percentage reduction for metabolic activity was observed at 3 months for 5% vancomycin vs. 5% dalbavancin (8.7% [0.0%–47.5%] and 43.9% [35.4%–95.3%], *p* = 0.044, respectively) (Figure 2).

### 2.3. Anti-Biofilm Activity of Antibiotics Loaded in Bone Cements

**Figure 3:**
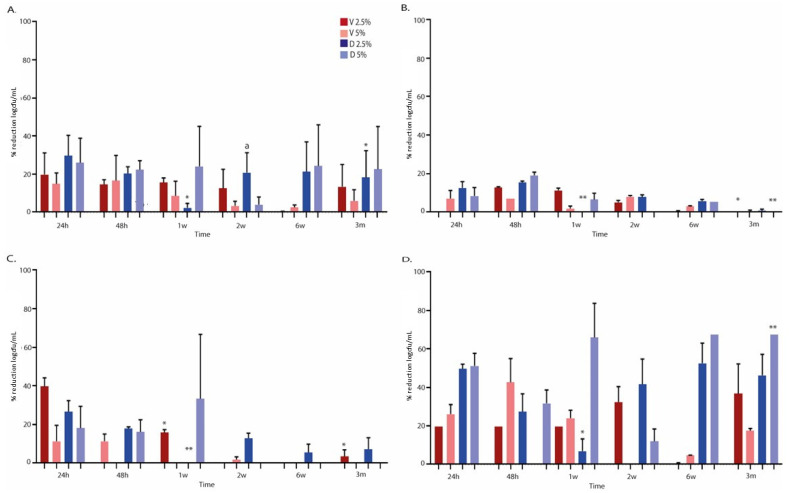
Mean percentage reduction in log cfu/mL in sonicated implants after being in contact with each antibiotic loaded in bone cement over a 3-month period. V, vancomycin; D, dalbavancin; h, hours; w, weeks; and m, months. (**A**). **Overall.** * Significant decrease between median log cfu/mL percentage reduction for 2.5% dalbavancin at 24 h vs. 1 w (*p* = 0.001) and 1 w vs. 3 m (*p* = 0.042). ^a^ Significant decrease between median log cfu/mL percentage reduction for 2.5% vancomycin and 2.5% dalbavancin at 2 w (*p* = 0.037). (**B**). **Methicillin-susceptible *Staphylococcus aureus* ATCC29213.** * Significant decrease between median log cfu/mL percentage reduction for 2.5% vancomycin at 1 w vs. 3 m (*p* = 0.037). ** Significant decrease between median log cfu/mL percentage reduction for 2.5% dalbavancin at 24 h vs. 1 w (0.037) and 24 h vs. 3 m (*p* = 0.046). (**C**). **Methicillin-resistant *Staphylococcus aureus* ATCC43300.** * Significant decrease between median log cfu/mL percentage reduction for 2.5% vancomycin at 24 h vs. 1 w (*p* = 0.05), 24 h vs. 3 m (*p* = 0.046), and 1 w vs. 3 m (*p* = 0.046). ** Significant decrease between median log cfu/mL percentage reduction for 2.5% dalbavancin at 24 h vs. 1 w (*p* = 0.037). (**D**). ***Staphylococcus epidermidis* ATCC35984.** * Significant decrease between median log cfu/mL percentage reduction for 2.5% dalbavancin at 24 h vs. 1 w (*p* = 0.046). ** Significant decrease in median log cfu/mL percentage reduction between 5% vancomycin vs. 5% dalbavancin at 3 m (*p* = 0.037).

The median percentage reduction in log cfu/mL at the end of the study period (3 months) did not change significantly for vancomycin or dalbavancin at either concentration used with respect to values at 24 h. In between periods, only a slight decrease in the median (IQR) log cfu/mL percentage reduction was detected between 24 h and 1 week and between 1 week and 3 months for 2.5% dalbavancin (25.4% [14.8%–48.4%] vs. 0.0% [0.0%–0.6%], *p* = 0.001; and 0.0% [0.0%–0.6%] vs. 2.9% [0.0%–35.7%], *p* = 0.042, respectively) (Figure 3A). In addition, when both antibiotics were compared, the median (IQR) percentage reduction in log cfu/mL at 2 weeks was significantly higher in 2.5% dalbavancin than in 2.5% vancomycin (4.5% [0.0%–23.0%] vs. 11.2% [8.1%–28.6%], *p* = 0.037).

**Figure 4:**
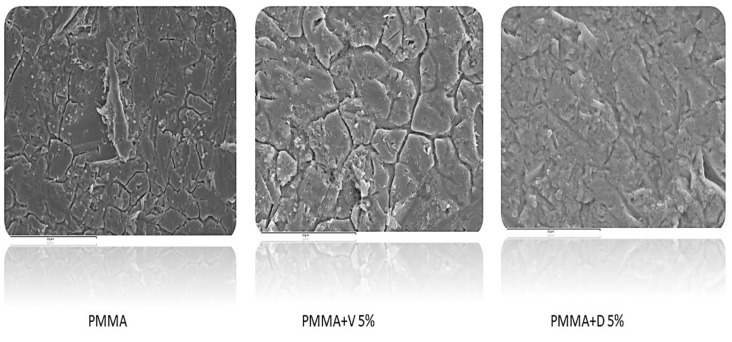
Scanning electron microscopy images of 24 h biofilms from methicillin-resistant *Staphylococcus aureus* ATCC43300 formed on implants in contact with 5% vancomycin and 5% dalbavancin loaded in bone cement at month 3. V, vancomycin; D, dalbavancin; and PMMA, polymethylmethacrylate. Images were taken at 2500× for PMAA+V5% and PMMA+D5% and at 1500× magnification for the PMMA control.

In MSSA in particular, we only observed a statistically significant decrease in the median (IQR) log cfu/mL percentage reduction between 24 h and 3 months in 2.5% dalbavancin (12.2% [7.0%–A], 0.0% [0.0%–NA], *p* = 0.046). In between periods, a significant decrease was also observed for 2.5% vancomycin at 1 week vs. 3 months and 2.5% dalbavancin at 24 h vs. 1 week (10.9% [9.3%–NA] vs. 0.0% [0.0%–NA], *p* = 0.037; and 12.2% [7.0%–NA] vs. 0.0% [0.0%–NA], *p* = 0.037) (Figure 3B). In MRSA, we only observed a significant decrease in median (IQR) log cfu/mL percentage reduction for 2.5% vancomycin between 24 h and 3 months (38.6% [32.3%–NA] vs. 0.0% [0.0%–NA], *p* = 0.046). In between periods, 2.5% vancomycin at 24 h vs. 1 week and 1 week vs. 3 months also decreased significantly (38.6% [32.3%–NA] vs. 16.6% [13.1%–NA], *p* = 0.05; and 16.6% [13.1%–NA] vs. 0.0% [0.0–NA], *p* = 0.046). Moreover, 2.5% dalbavancin decreased significantly in terms of the median (IQR) log cfu/mL percentage reduction between 24 h and 1 week (25.4% [17.3%–NA] vs. 0.0% [0.0%–NA], *p* = 0.037) (Figure 3C). In *S. epidermidis*, a significant decrease in the median (IQR) log cfu/mL percentage reduction between 24 h and 1 week was only observed for 2.5% dalbavancin (52.0% [44.8%–NA] vs. 1.2% [0.0%–NA], *p* = 0.046). In addition, the median (IQR) log cfu/mL percentage reduction decreased significantly for 5% vancomycin compared to 5% dalbavancin at 3 months (17.4% [15.5%–NA] vs. 67.5% [67.5%–67.5%], *p* = 0.037) (Figure 3D). This finding was also corroborated by the images obtained using scanning electron microscopy (SEM), as shown in Figure 4, where we observed a greater reduction in the presence of MRSA in bone cement loaded with 5% dalbavancin than in bone cement loaded with 5% vancomycin.

## 3. Discussion

Although dalbavancin is approved for the treatment of skin and soft tissue infections, successful outcomes have been widely reported in patients with PJI [14,15]. In addition, the drug has been shown to be cost-effective [16].

The success of dalbavancin in the treatment of PJI is related mainly to the eradication of bacterial biofilms, against which dalbavancin is highly active [17]. In the study by Pfaller et al. [18], 800 *S. aureus* strains from United States and European hospitals isolated between 2011 and 2016 were tested against several antimicrobials, and the minimum inhibitory concentration (MIC) for dalbavancin was at least eight-fold lower than for the other agents. Sivori et al. [19] showed that the minimal biofilm eradication concentration (MBEC_90_) values for dalbavancin were significantly lower than those of linezolid and vancomycin in 32 MRSA strains tested. The authors also demonstrated that its activity was affected by an increased concentration of extracellular DNA in the biofilm matrix.

Moreover, in an in vitro time-kill kinetics model, dalbavancin was tested against *S. aureus* and *S. epidermidis* biofilms for up to 7 days and was shown to be more active than vancomycin [20].

Therefore, based on these data and on the demonstration that it had excellent bone distribution, dalbavancin loaded in bone cement is an attractive approach for the treatment of PJI [21]. As we demonstrated in a previous study, dalbavancin showed good elution capacity when loaded in bone cement, remaining for almost 14 days [13]. In the present study, we confirmed that eluted dalbavancin from both the 2.5% and the 5% formulations maintained concentrations above 2 µg/mL for up to 3 months. In addition, despite specific fluctuations between periods, dalbavancin was sufficiently efficacious to reduce 24 h staphylococcal biofilms, as the median percentage reduction in log cfu/mL at 3 months did not change significantly in vancomycin or in dalbavancin at either of the concentrations used with respect to values at 24 h. Specifically, the significant differences between median (IQR) log cfu/mL percentage reduction in 5% vancomycin and 5% dalbavancin at 3 months were observed in *S. epidermidis* (17.4% [15.5%–NA] vs. 67.5% [67.5%–67.5%], *p* = 0.037). It was also important to highlight that the median (IQR) percentage reduction in metabolic activity of staphylococcal biofilms exposed to eluted antibiotics was significantly lower for 5% vancomycin than for 5% dalbavancin (8.7% [0.0%–47.5%] vs. 43.9% [35.4%–95.3%], *p* = 0.044, respectively).

The significant differences in release profiles between the two antibiotics could result from the high solubility and smaller size of the vancomycin molecule with respect to dalbavancin, even though both are considered very large molecules. It seems that vancomycin was degraded at the beginning of the study period, as reflected in the reduced concentration, and then, owing to the drug’s high elution capacity, the concentration began to accumulate. In contrast, dalbavancin reached its highest concentration at 2 weeks before beginning to degrade, as there was no further elution; however, its concentration remained constant over time. These data differ from those of our previous study because the concentration analyzed was cumulative [13]. Either way, although the concentration of dalbavancin recovered during elution is lower than that of vancomycin, it is higher than the MIC against staphylococci.

While our findings are promising, we were unable to completely eradicate biofilm. This is consistent with the results obtained in the in vivo mouse model of Silva et al. [22], who reported that, although administering intraperitoneal dalbavancin for 14 days decreased cfu/gr and cfu/mL in both the tibia and the implant, there were still signs of biofilm-induced infection. Therefore, new approaches are needed to improve dalbavancin activity in bone cements, such as combining it with rifampicin, as reported elsewhere [23,24,25].

One of the main limitations of our study is that we used an in vitro static biofilm model, which may not represent the real clinical scenario. In fact, as we tested loaded bone cements against biofilm—and not against planktonic cells—as prophylaxis, we were too demanding in terms of efficacy. In addition, the behavior of sessile cells may differ completely from that of planktonic cells subjected to factors other than the antibiotic concentration, which could explain the discordance between the percentage reduction in metabolic activity or cfu counts at the different antibiotic concentrations and the concentrations detected at specific elution times. In addition, we analyzed the drugs using different instruments because, although HPLC is generally the standard method for analyzing compounds in the pharmaceutical industry, we did not obtain a good separation pattern with dalbavancin. Therefore, we preferred to analyze the samples with MS. Future in vivo or dynamic models mimicking PJI are needed to corroborate our findings, for example, by increasing monitoring for up to 6 months. Cytotoxicity experiments using dalbavancin-loaded bone cements are also essential.

## 4. Materials and Methods

This study was carried out in a tertiary teaching hospital in Madrid, Spain.

### 4.1. Preparation of the Antibiotic-Loaded Bone Cements

Palacos^®^ R bone cement (Heraeus Medical GmbH, Wehrheim, Germany) was mixed manually with either vancomycin or dalbavancin powder at concentrations of 2.5% (1 g of antibiotic per 40 g of cement) and 5% (2 g of antibiotic per 40 g of cement). Discs measuring 3 × 6 mm were prepared using a mold (Supplementary Figure S1). These were then incubated in phosphate-buffered saline (PBS) at 37 °C under shaking (150 rpm) for 24 h, 48 h, 1 week, 2 weeks, 6 weeks, and 3 months. All experiments were performed in triplicate, and negative controls were included. At each timepoint, 1 mL aliquots of the eluate were obtained and frozen for the following analyses: high-performance liquid chromatography (vancomycin), mass cytometry (dalbavancin), and anti-biofilm activity against 24 h biofilms in 96-well plates. Likewise, discs were rinsed with PBS for the anti-biofilm experiments with steel implants during each study period (Figure S1).

### 4.2. Vancomycin Analysis

Samples were analyzed using HPLC with a Varian Prostar 230 Solvent Delivery Module, Varian Prostar autosampler 410, and Varian Prostar 310 UV–visible detector (Varian, Palo Alto, CA, USA). Peak integration was analyzed using Galaxie software (version 1.9). Vancomycin was analyzed using a Nucleosil C18 column (250 mm × 4.6 mm, 5 μm). The mobile phase consisted of 50 mM ammonium phosphate and acetonitrile (92:8, *v*:*v*) (final pH of 2.2) and was pumped at a flow rate of 0.7 mL/min. The injection volume of the sample was 40 µL. The column temperature was maintained at 25 °C, and the detector was set at 205 nm.

### 4.3. Dalbavancin Analysis

Samples were analyzed using LC-ESI-QQQ-MS (LC-8030 Shimadzu, Manchester, UK). Dalbavancin was analyzed using a Phenomenex Gemini C18 column (110 A 150 mm × 2 mm, 5 μm). The gradient mode consisted of the following: 5% Phase B—7 min; 95% Phase B—8 min; 95% Phase B—8.5 min; and 5% Phase B. Phase A consisted of H_2_O + 0.1% formic acid and Phase B of CAN. The phase was pumped at a flow rate of 0.4 mL/min, and the injection volume of the sample was 40 µL. The run time was 10 min, and the MRM transitions for D were as follows: Quantifier (*m*/*z*), 909.1 > 340.0 [CE, -36]; Qualifier (*m*/*z*), 909.1 > 730.5 [CE, -26]).

The elution tests were performed in triplicate.

### 4.4. Anti-Biofilm Activity of Eluted Antibiotics against 24 h Biofilms in 96-Well Plates According to the Reduction in Metabolic Activity at Each Timepoint

At each study period, 24 h biofilms of each species were formed on 96-well plates by adding 100 µL of a 10^8^ cfu/mL (0.5 McFarland) bacterial suspension prepared in broth media (TSB for S. aureus and TSB+1% glucose for *S. epidermidis*) previously incubated at 37 °C for 24 h under agitation. Plates were then incubated for 24 h at 37 °C, and 3 gentle PBS washes were performed. Once the biofilms on the wells were dried, 100 µL of eluted antibiotics or PBS (in the positive control wells) from each timepoint was added, and plates were incubated for 24 h at 37 °C. Then, 3 gentle PBS washes were performed, and once the wells were dried, 100 µL of XTT (with menadione at 1:1000) was added, and plates were incubated for 2 h at 37 °C in darkness. After incubation, the content of the wells was transferred to a new plate, and absorbance was measured in a spectrophotometer at 490 nm (Figure S1). We calculated the percentage reduction in metabolic activity as follows:

(1) $$\left[1-\left(\frac{abs\;treated\;well}{abs\;positive\;control}\right)\right]\times 100$$

where abs is the absorbance value at 490 nm.

Experiments were only performed once.

### 4.5. Anti-Biofilm Activity of Antibiotics Loaded in Bone Cements against 24 h Biofilms in Steel Implants According to log cfu/mL Reduction at Each Timepoint

At each study timepoint, 24 h biofilms of each species were formed on steel implant discs measuring 3 × 6 mm (previously sterilized with ethanol followed by autoclave) by adding 1 mL of a 10^8^ cfu/mL (0.5 McFarland) bacterial suspension prepared in broth media (TSB for S. aureus and TSB + 1% glucose for *S. epidermidis*), previously incubated at 37 °C for 24 h with shaking, to a glass tube. Tubes were then incubated for 24 h at 37 °C with shaking, and 3 gentle PBS washes were performed. Once the biofilms on the implant were dried, both the steel disc and the antibiotic-loaded cement bone disc (or without antibiotics for the positive control) for each timepoint were placed together in a glass tube with 1 mL of PBS. The tubes were incubated for 24 h at 37 °C under agitation. Then, 3 gentle PBS washes were performed, and steel implant discs were sonicated for 10 min at 40 kHz in 1 mL of PBS. Serial dilutions of the sonicate were performed, and 100 µL was cultured onto blood agar plates (Figure S1). Colony-forming units were counted, and data were expressed as log cfu/mL. We calculated the percentage reduction in log cfu/mL as follows:

(2) $$\left[1-\left(\frac{\log cfu\times {\mathrm{mL}}^{-1}\;antibiotic-loaded\;cement}{\log cfu\times {\mathrm{mL}}^{-1}\;non–antibiotic-loaded\;cement}\right)\right]\times 100$$

Experiments were performed in triplicate.

This procedure (until the sonication step) was performed twice in month 3 in order to analyze implant biofilm structure using scanning electron microscopy (SEM).

### 4.6. Scanning Electron Microscopy Analysis of Implant Discs at Month 3

Once the implant discs were tested with 3-month incubated bone cement discs and 3 gentle PBS washes were performed, they were transferred to a new tube with 10% glutaraldehyde to be further analyzed using SEM. Prior to visualization under SEM, the samples were dehydrated using increasing concentrations of ethanol.

### 4.7. Statistical Analysis

We used the *t* test to compare quantitative variables between groups.

Statistical significance was set at *p* < 0.05 for all the tests. The statistical analysis was performed using IBM SPSS Statistics for Windows, Version 21.0 (IBM Corp, Armonk, NY, USA).

We compared the median concentration for each antibiotic between the following periods: 24 h vs. 1 week, 24 h vs. 3 months, 1 week vs. 3 months, and 2 weeks vs. 3 months. We compared the median percentage reduction in log cfu/mL and the median percentage reduction in metabolic activity for each antibiotic between the first and last timepoints (24 h vs. 3 months) and between the following intermediate periods: 24 h vs. 1 week and 1 week vs. 3 months. Comparisons between vancomycin and dalbavancin were only performed at 2 weeks and 3 months for each concentration used. The analysis was performed for all microorganisms together and for each species (MSSA, MRSA, and *S. epidermidis*), except for the metabolic activity analysis, in which no triplicates were performed. Therefore, the median percentage reduction was only compared for all microorganisms together, but not for each species separately.

Comparisons of the statistical significance between groups are detailed in the figure legends, which highlight only those in which a significant reduction was observed.

## 5. Conclusions

The concentration of dalbavancin decreased significantly from 2 weeks of incubation but maintained its anti-biofilm activity for up to 3 months (>50% reduction in metabolic activity). The concentration of vancomycin decreased significantly at 1 w before increasing gradually. The anti-biofilm activity of vancomycin was significantly lower than that of dalbavancin.

Dalbavancin loaded in bone cement seems a promising alternative for the treatment of PJI. Release is constant, and the results are better than for vancomycin at both the 2.5% and the 5% concentrations. Dalbavancin also has a lower MIC_90_ than vancomycin (0.06 mg/L vs. 2 mg/L, respectively) and a longer half-life.

## Data Availability

Not applicable.

## Supplementary Materials

The following supporting information can be downloaded at https://www.mdpi.com/article/10.3390/antibiotics12091445/s1. Figure S1: Laboratory procedure. Figure S2: Elaboration of antibiotic-loaded PMMA bone cement discs.
