# Supplementary material for: In Vitro Efficacy of Dalbavancin as a Long-Acting Anti-Biofilm Agent Loaded in Bone Cement
Source: Antibiotics (Basel). 2023 Sep 13;12(9):1445. doi: 10.3390/antibiotics12091445 (PMC10525811; doi:10.3390/antibiotics12091445)
Supplement: Supplementary file 1 [file antibiotics-12-01445-s001.zip › antibiotics-2502467-supplementary.pdf]

Supplementary Materials

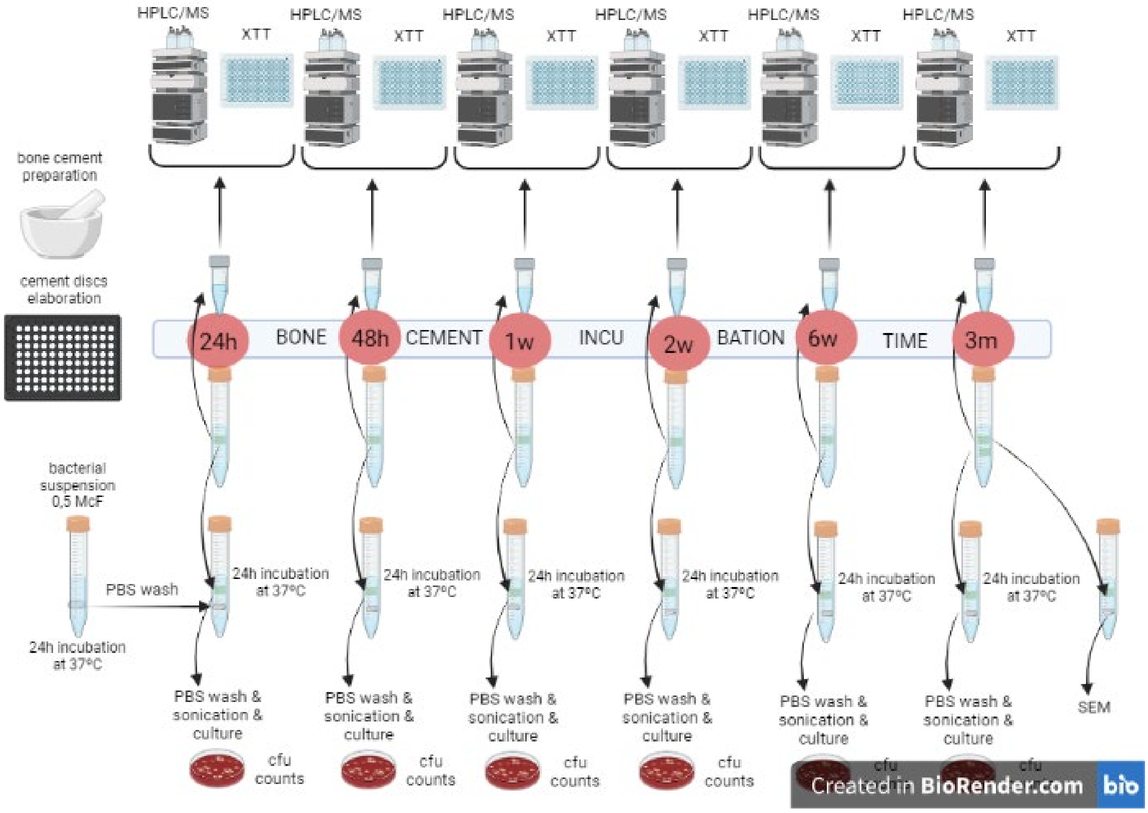

Figure S1: Laboratory procedure

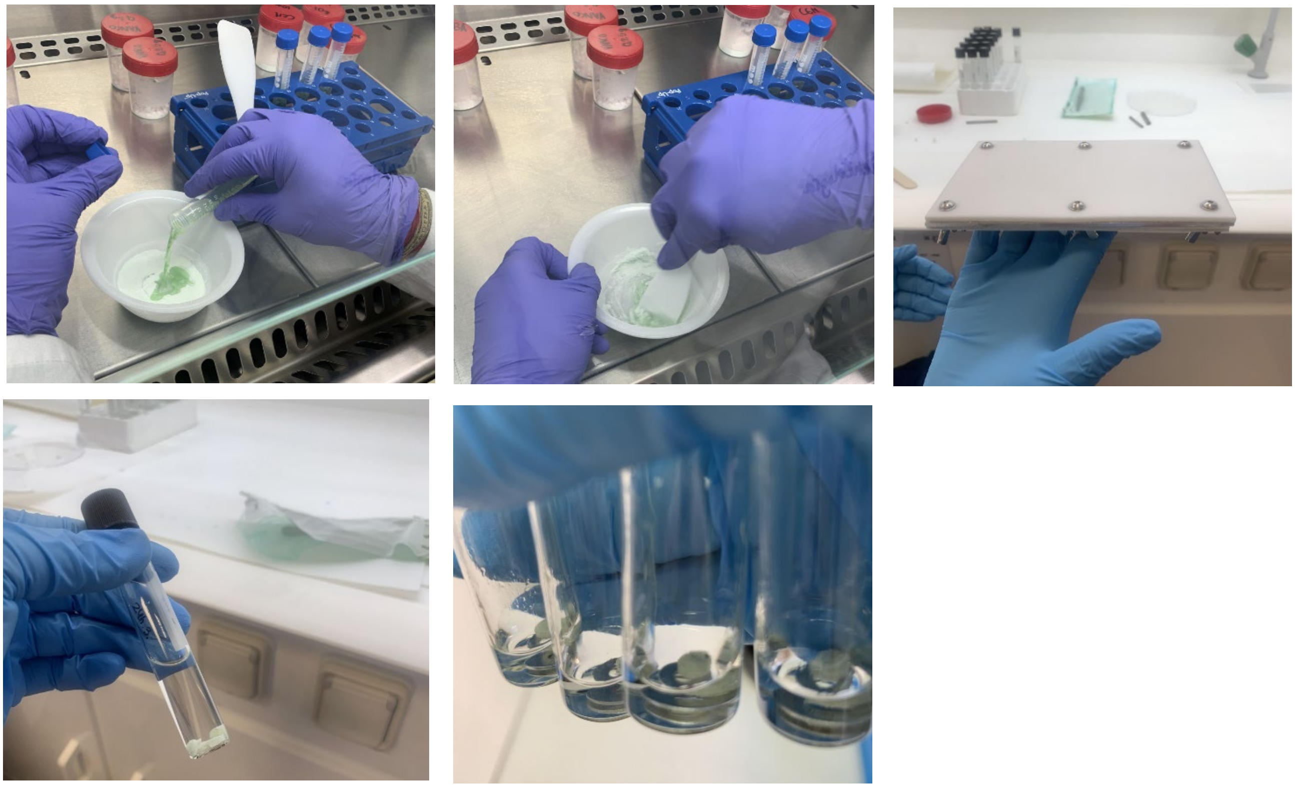

Figure S2. Elaboration of antibiotic loaded PMMA bone cement discs
